# The Optimized Homogenization Process of Cast 7Mo Super Austenitic Stainless Steel

**DOI:** 10.3390/ma16093438

**Published:** 2023-04-28

**Authors:** Runze Zhang, Jinshan He, Shiguang Xu, Fucheng Zhang, Xitao Wang

**Affiliations:** 1 Collaborative Innovation Center of Steel Technology, University of Science and Technology Beijing, Beijing 100083, China; 2 State Key Laboratory of Metastable Materials Science and Technology, Yanshan University, Qinhuangdao 066004, China; 3 Shandong Provincial Key Laboratory for High Strength Lightweight Metallic Materials, Advanced Materials Institute, Qilu University of Technology (Shandong Academy of Science), Jinan 250353, China

**Keywords:** super austenitic stainless steel, homogenization, Cerium, Manganese

## Abstract

Super austenitic stainless steels are expected to replace expensive alloys in harsh environments due to their superior corrosion resistance and mechanical properties. However, the ultra-high alloy contents drive serious segregation in cast steels, where the σ phase is difficult to eliminate. In this study, the microstructural evolution of 7Mo super austenitic stainless steels under different homogenization methods was investigated. The results showed that after isothermal treatment for 30 h at 1250 °C, the σ phase in steels dissolved, while the remelting morphologies appeared at the phase boundaries. Therefore, the stepped solution heat treatment was further conducted to optimize the homogenized microstructure. The samples were heated up to 1220 °C, 1235 °C and 1250 °C with a slow heating rate, and held at these temperatures for 2 h, respectively. The elemental segregation was greatly reduced without incipient remelting and the σ phase was eventually reduced to less than 0.6%. A prolonged incubation below the dissolution temperature will lead to a spontaneous compositional adjustment of the eutectic σ phase, resulting in uphill diffusion of Cr and Mn, and reducing the homogenization efficiency of ISHT, which is avoided by SSHT. The hardness reduced from 228~236 Hv to 220~232 Hv by adopting the cooling process of “furnace cooling + water quench”. In addition, the study noticed that increasing the Ce content or decreasing the Mn content can both refine the homogenized grain size and accelerate diffusion processes. This study provides a theoretical and experimental basis for the process and composition optimization of super austenitic stainless steels.

## 1. Introduction

7Mo super austenitic stainless steel (SASS) is the highest-alloyed austenitic stainless steel with more than 50% alloying elements, including molybdenum and nickel. It possesses stronger corrosion resistance (PREN ≥ 40) and mechanical properties than common stainless steels [1]. Compared with nickel-based alloys, 7Mo SASS has significant advantages of cost-effectiveness and rivaled-performance, which can partially replace the application of expensive alloys in harsh environments [2]. However, the formation of the σ phase is difficult to control due to the strong tendency of elemental segregation. The σ phase is a brittle phase that arises as a eutectic phase in SASSs [3]. An excessive σ phase will cause cracking during processing [4,5] and reduce corrosion resistance [6]. In order to reduce the influence of the σ phase on hot processing and application, the σ phase can be reduced by regulating the alloy composition [3,7,8,9] or by adopting homogenization treatment [10]. However, the dissolution temperature of the σ phase increases with the increasing alloying contents in steels, to pose a challenge in the homogenization process [11]. 

The traditional homogenization process for steels is the isothermal solution heat treatment (ISHT), with the main processes of heating → holding → rapid cooling. In 304 stainless steel (18Cr-8Ni), homogenization can be achieved by holding the steel at 1050~1150 °C for 0.17 h [12,13]. The first-generation SASS 904 L (20Cr-23Ni-4Mo) needs to be held at 1100~1150 °C for 4~8 h. Until 654 SMO (25Cr-20Ni-7Mo), where the temperature is increased to 1280 °C and it need to be held for more than 30 h [14,15]. At the same time, as the Mo contents increase further, the dissolution temperature of the eutectic σ phase gradually increases, accompanied by the lower liquidus temperature of alloys [16,17]. Therefore, higher-alloy contents providing excellent overall performance pose a significant challenge to subsequent thermal processing.

The stepped solution heat treatment (SSHT) is widely used in superalloys, which can effectively increase the melting point of inter-dendrites by holding the steel slightly below the overburning temperature [18]. For this method, a slow heating rate is used as part of the homogenization [19]. Due to the significant differences between SASSs and common stainless steels, SASSs have similar characteristics with the superalloys, including: (1) high alloying contents (>50%) with serious element segregation; (2) the eutectic phase with high dissolution temperature; (3) the heat treatment window is extremely narrowed. For these reasons, optimizing the homogenization parameters of SASSs based on the typical SSHT is possible and has a prospect of application. 

Alloying elements can also influence the homogenization efficiency by changing the as-cast microstructure. For example, alloying modifier can increase crystal defects to accelerate the atomic diffusion, while the addition of refractory elements with a slow diffusion rate will greatly increase the homogenization time [17]. Typical refiners in steels are rare earth elements, which have been confirmed as helpful elements in reducing the second phase [7,20,21,22]. The austenite-forming element Mn has also been studied in high-alloyed steels for reducing the element segregation tendency [7,23]. However, the Mn content in SASSs is controlled below 3 wt.% mostly, and the application of rare earth elements in SASSs is still in the initial stage. The effect of Ce and Mn elements on SASSs still needs to be explored. 

In this study, in order to reduce the segregation and the σ phase, based on the 654SMO, we developed 7Mo SASSs by adding Ce elements and raising Mn elements. Thermodynamic calculations show the dissolution temperature of the σ phase in 7Mo SASSs over 1270 °C, while the remelting temperature is about 1250 °C. Therefore, it is very difficult to dissolve the σ phase completely. To develop a suitable homogenization process—by adopting ISHT and SSHT, exploring the evolution of the second phase and element segregations—we find that SSHT can shorten the homogenization time effectively and optimize the microstructure. The effect of Ce and Mn elements on the homogenization process was also explored. This study applies the SSHT innovatively to austenitic stainless steels to provide theoretical guidance for the production and development of 7Mo SASSs. It also establishes the foundation for the composition optimization of super austenitic stainless steels.

## 2. Materials and Methods

**Figure 1:**
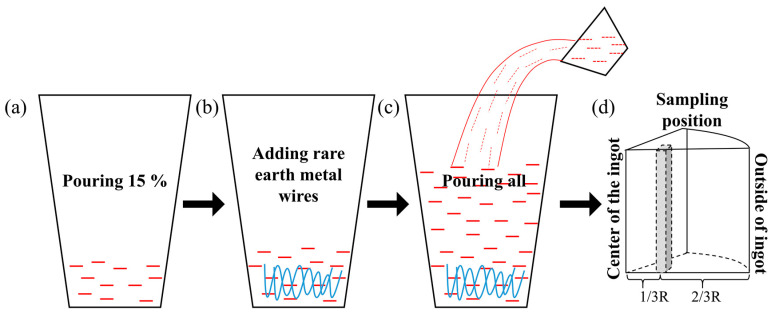
(**a**–**c**) 7Mo super austenitic stainless steel smelting process; (**d**) Sampling location.

The 7Mo SASS ingots were all provided by Taiyuan Iron & Steel Group Co. LTD of China. Test steels for each composition were smelted in a vacuum induction furnace (VIM). The smelting process is shown in Figure 1: pouring 15% liquid steel into the VIM, feeding rare earth wire with full stirring, then pouring the remaining liquid steel and solidifying. The sampling location is shown in Figure 1d, from the edge to 2/3R of the center. The chemical composition of 7Mo SASSs in the experiments are shown in Table 1. The nitrogen content of the samples was determined by oxygen, nitrogen and hydrogen analyzers (TCH-600). The content of rare earth Ce in the steel was measured by inductively coupled plasma mass spectrometry (ICP-MS, TQ-ICP-MS)). Other alloying elements in the steel were detected by inductively coupled plasma emission spectrometry (ICP-OES, Optima 5300 DV).

**Figure 2:**
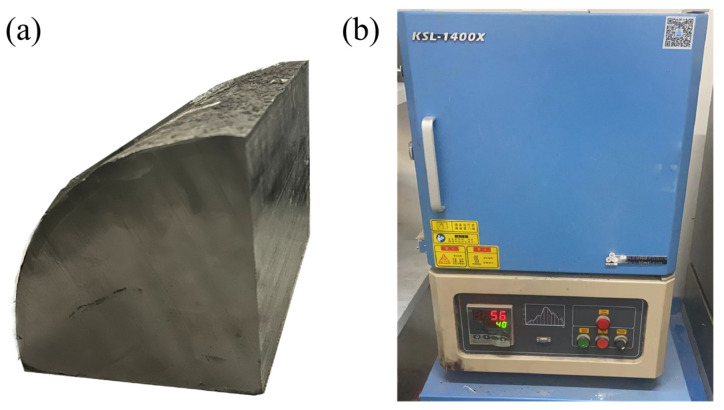
(**a**) the ingot for the experiment; (**b**) the muffle furnace (KSL-1400X).

Each of the three samples was cut into 10 mm × 10 mm × 10 mm. The actual ingot we used is shown in Figure 2a. All homogenization processes were performed in a muffle furnace (KSL-1400X) with a refractory brick door as shown in Figure 2b. In order to prevent high-temperature oxidation, the samples were protected by argon gas at high temperature. To minimize the temperature fluctuation, B-type thermocouples were used to control the temperature.

**Figure 3:**
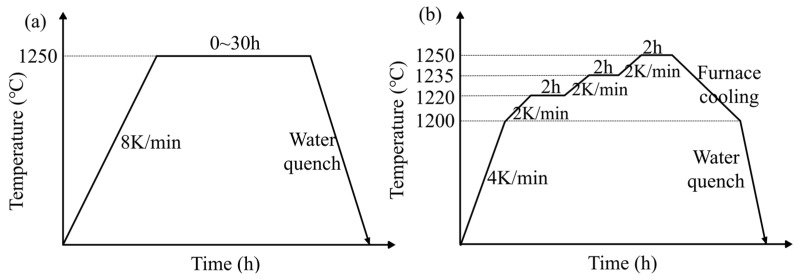
(**a**) ISHT parameters; (**b**) SSHT parameters.

The parameters of the ISHT and SSHT are given in Figure 3. For ISHT, the samples were homogenized at 1250 °C and held for 30 h, followed by water quench. For SSHT, the samples were heated at 4 K/min to 1200 °C with the furnace held for 2 h, followed by heating at 2 K/min to 1235 °C held for 2 h, then heated at 2 K/min to 1250 °C for 2 h. At each stage, the “furnace cooling + water quench” was used to obtain the homogenized samples.

**Figure 4:**
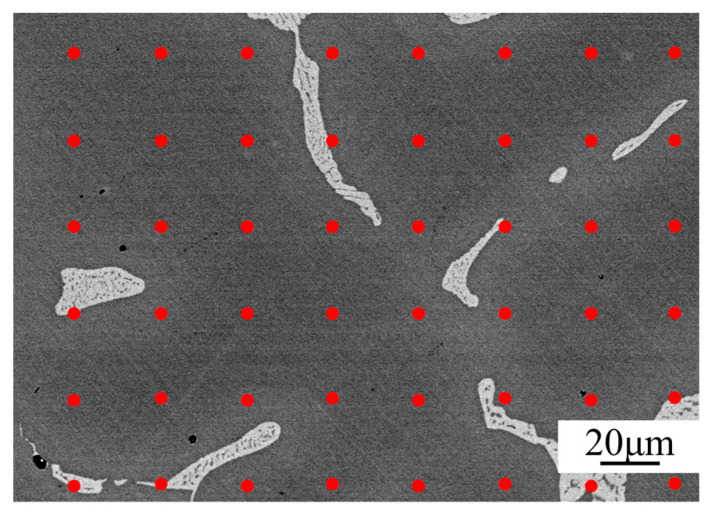
Schematic diagram of component distribution in dot-matrix method: a large number of points are evenly selected on the sample as shown by the red dots.

Before observation, samples were polished with 50 #~2000 # sandpaper and mechanically polished with 1.5~2.5 diamond polishing paste. The distribution of the second phase was observed by field emission scanning electron microscopy (SEM, SM-7001F) with a backscattering probe (BSE). As shown in Figure 4, the σ phase in the polished sample appears bright-white under SEM-BSE, and the morphology of inter-dendrites can be observed at the same time. X-ray diffraction (XRD, Rigaku TTR3) was used for the identification of phases with a step of 0.02°, a scan time of 3 s per step, and a scan angle of 30°~120°. After the homogenization, the grain boundaries were observed and counted by an optical microscope (OM, Axio Imager M2m). The electrolytic erosion had parameters of 10% ethanol hydrochloric acid at 7 V for 40 s.

The electron probe X-ray micro-analyzer (EPMA, model JXA-8530F) was used for measuring the distribution of elements and the samples were polished after grinding. Since the dendritic morphology after homogenization is inconspicuous, the element distribution can be determined using the dot-matrix method by EPMA [24], as shown in the Figure 4. The homogenized samples were observed at the same magnification as cast samples. A large number of points were selected within the area, and each point was detached and classified.

Vickers hardness (VTD512) was used to test the hardness of each sample. Before the test, they were grounded and polished. Twenty points were randomly selected in each sample center, and the load was set as 500 g and kept for 15 s. Finally, the average value of the tests were taken.

All thermodynamic calculations in this study were performed using the Thermo-calc software 2023a with TCFE10 database. DICTRA dynamics calculation was used to calculate the distribution of elements at the inter-dendrite and the σ/γ phase interface during homogenization. The database was MOBFE4. 

To solve the homogenization problem, the single-phase model was adopted. Because of the symmetry of the as-cast microstructure, the three-dimensional diffusion process of alloy was simplified into one dimensional problem by using the plate model. According to the average size of equiaxed crystal measured by experiments, the minimum diffusion unit is taken as 1/2 of the size of equiaxed crystal. Considering the accuracy and efficiency of the simulation, the diffusion element was divided into 50 nodes. The initial composition distribution was defined based on the results of the EPMA tests. Finally, the time and temperature conditions of the simulation homogenization were set. 

In order to describe the distribution of elements in the front of the interphase boundary during the dissolution process, the plate model was used to divide two-phase regions. The minimum diffusion unit of the σ phase was 5 μm, and the minimum diffusion unit of austenite was 1/2 length of isometric crystal size of several steels. The initial composition distribution was defined according to the EPMA results. The σ phase diffusion element was divided into 10 nodes and the austenitic diffusion element into 50 nodes.

## 3. Results

### 3.1. As-Cast Microstructure

**Figure 5:**
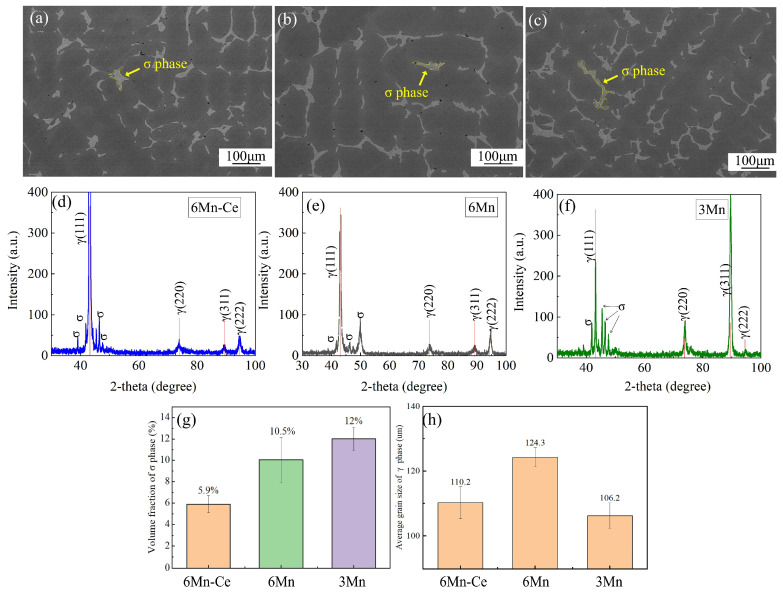
(**a**–**c**) the microstructure of 6Mn-Ce, 6Mn and 3Mn under SEM-BSE; (**d**–**f**) the XRD results of 6Mn-Ce, 6Mn and 3Mn; (**g**,**h**) summary of the volume fraction of σ phase and the average size of isotropic crystals.

Figure 5 shows the microstructures of 6Mn-Ce (Figure 5a), 6Mn (Figure 5b) and 3Mn (Figure 5c) under SEM-BSE. The second phase appears bright-white in color with a coral-like morphology distributed at inter-dendrites, showing a typical eutectic morphology and a high-level element enrichment. The phases were analyzed by XRD as shown in Figure 5d–f. It can be seen that the as-cast sample consisted of the σ + γ phases, which is consistent with previous studies [25]. By comparing the XRD pattern of the three steels, there are multiple crystalline σ phase diffraction peaks in the 6Mn-Ce and 3Mn steels, which should be caused by the finer austenite dendrites and more dispersed σ phase. Meanwhile, the (111) peak of austenite in 6Mn-Ce was significantly wider, and this phenomenon is mainly due to the lattice distortion caused by the increased Mn and Ce content in the steel. The volume fractions of the σ phase and average grain size were counted in Figure 5g,h. The volume fractions of the σ phase in 6Mn-Ce, 6Mn and 3Mn steels were 5.9%, 10.5% and 12.0%, respectively. 6Mn-Ce, 6Mn -0.001Ce and 3Mn had an average isometric crystal size of 110.2 μm, 124.3 μm and 106.2 μm, respectively. It is obvious that increasing the Ce content or reducing the Mn content is beneficial for refining the microstructure.

### 3.2. Determination of the Heating Temperature

**Figure 6:**
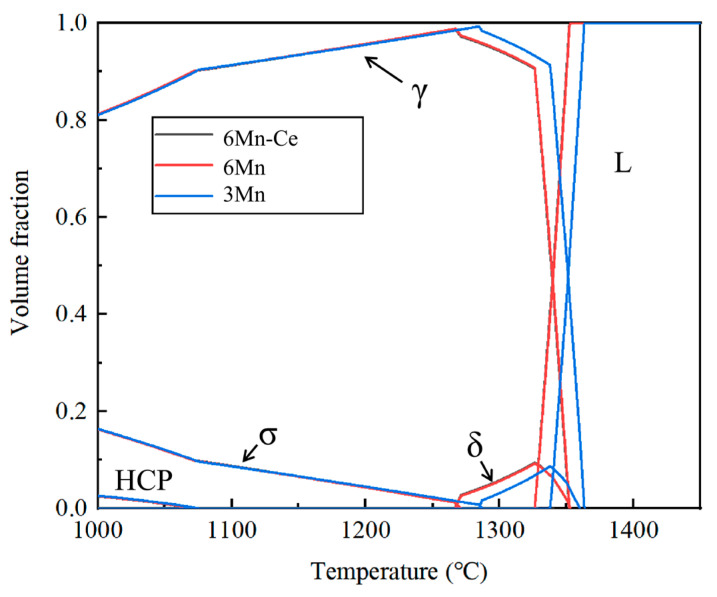
The equilibrium diagrams of 6Mn-Ce, 6Mn and 3Mn.

The phase transition temperature is crucial to guiding the heat treatment and thermal processing. The equilibrium phase diagrams of 6Mn-Ce (black line), 6Mn (red line) and 3Mn (blue line) obtained by the Thermo-calc thermodynamic calculation are shown in Figure 6. The predicted temperatures are concluded in Table 2. T_σ_ is the temperature at which the σ phase dissolved completely. T_δ_ and T_L_ are the lowest temperatures at which the δ phase and the liquid appeared, respectively. In both 6Mn-Ce and 6Mn, the σ phase dissolved at 1267 °C, while the δ phase formed at 1268 °C and remained stable until the liquid formed. It can be seen that for 6Mn SASSs with different Ce contents, the single-austenite region was only 1 °C, and the temperature difference between T_L_ and T_σ_ was only 59 °C. For 3Mn steel, the σ phase was completely dissolved at 1279 °C, and the δ phase appeared at 1285 °C until the liquid phase formed, where the single-austenite temperature range was also only 6 °C and the temperature window between T_L_ and T_σ_ was 59 °C. Therefore, increasing the Mn content narrows the single-austenite region of 7Mo SASSs, while Ce has little effect on it.

The Gulliver-Scheil calculation [26] was further used to predict the melting point between dendrites based on the EPMA results, and is denoted as T_LL_ in Table 3. As can be noticed, the maximum temperature for the homogenization of 6Mn steel with different Ce content should be lower than 1250 °C, while the ultimate temperature for 3Mn steel was 1260 °C. According to Figure 6, the volume fractions of the σ phase at 1250 °C under equilibrium in 6Mn-Ce, 6Mn and 3Mn were 0.206%, 0.199% and 0.0215%, respectively. A study showed that the corrosion resistance of steels is hardly affected when the volume fraction of the σ phase is below 0.6% [27]. In order to facilitate the comparison of the effects of Ce and Mn, 1250 °C was chosen as the extreme temperature for the homogenization process.

### 3.3. The Process of ISHT and SSHT

**Figure 7:**
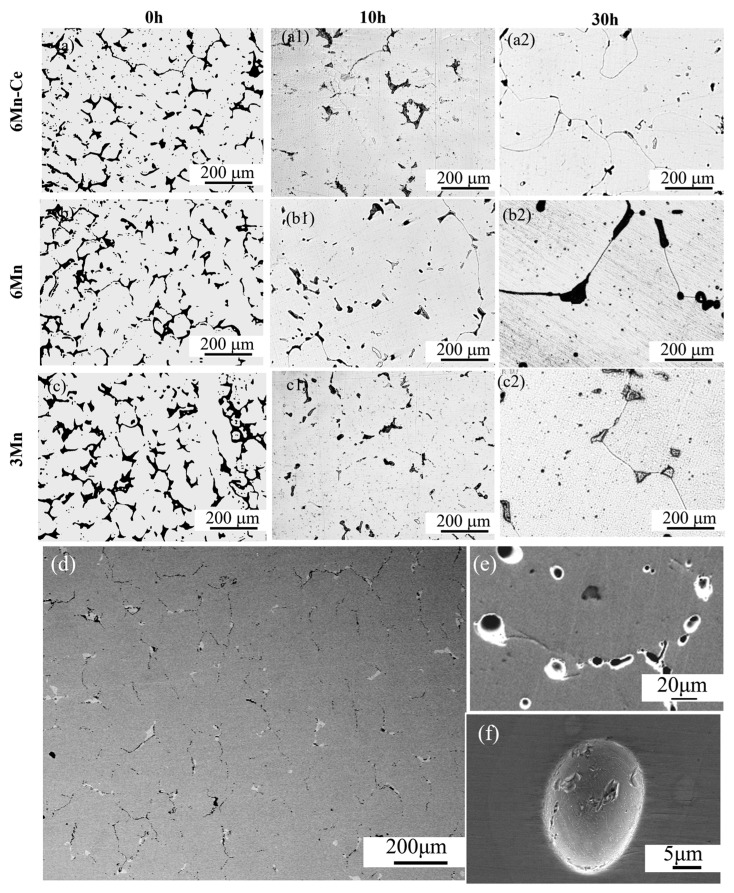
The microstructural evolution of (**a**–**a2**) 6Mn-Ce, (**b**–**b2**) 6Mn and (**c**–**c2**) 3Mn during ISHT; (**d**,**e**) remelting holes after ISHT; (**f**) the remolten ball.

Figure 7a–c shows the microstructural evolution of 6Mn-C, 6Mn and 3Mn during the ISHT. According to the morphological observation, the σ phase in the three steels gradually transforms from a coral-like shape to a regular shape, and the dendritic morphology also disappears with the extension of time. After holding for 30 h, the σ phase in the three steels almost completely dissolved and equiaxed crystals formed. Further analysis of the microstructure at 1250 °C as shown in Figure 7d–f, which contains a large number of regular-shape holes, appeared at the interface of the σ phase. From Figure 7e, a ring exists around these holes, which is a typical remelting morphology. Figure 7f shows a remolten ball with a unique patterned surface in the homogenized sample. The appearance of these defects indicates that incipient remelting occurred after the ISHT [28].

**Figure 8:**
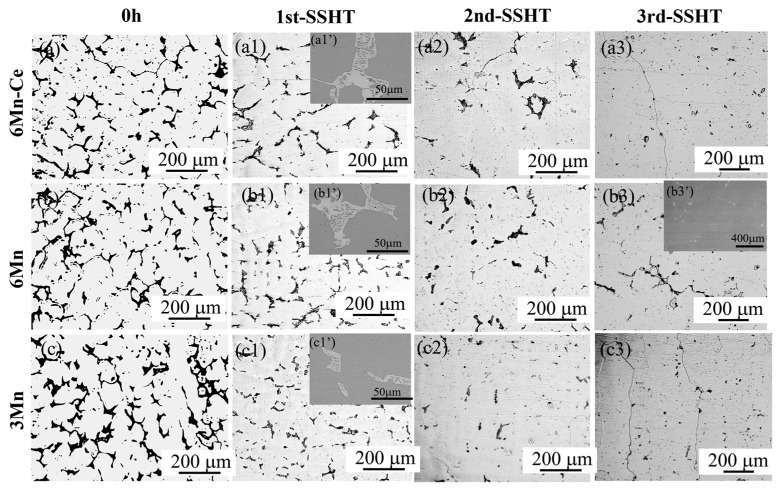
Microstructure evolution of steels during SSHT: (**a**–**a3**) 6Mn-Ce, (**b**–**b3**) 6Mn and (**c**–**c3**) 3Mn; (**a1’**,**b1’**,**c1’**) show the eutectic σ phase in three steels without remelting after 1 st-SSHT; (**b3’**) indicates that the 6Mn steel with the lowest melting point remained a small amount of σ phase without remelting after 3rd-SSHT.

The microstructure evolution during SSHT was subsequently observed, as shown in Figure 8. After the 1st-SSHT, a large number of the σ phase remained in all samples, and the size of the γ phase inside the coral-like σ phase expanded, as shown in Figure 8a1’–c1’. Until the completion of the 3rd-SSHT, the σ phase was almost dissolved in 6Mn-Ce and 3Mn steels. Further observation of the 6Mn steel after the 3rd-SSHT without remelting holes, as shown in Figure 8b3’, indicated that SSHT can effectively alleviate the remelting of the alloy.

### 3.4. Dissolution of σ Phase and Diffusion of Elements during ISHT and SSHT

**Figure 9:**
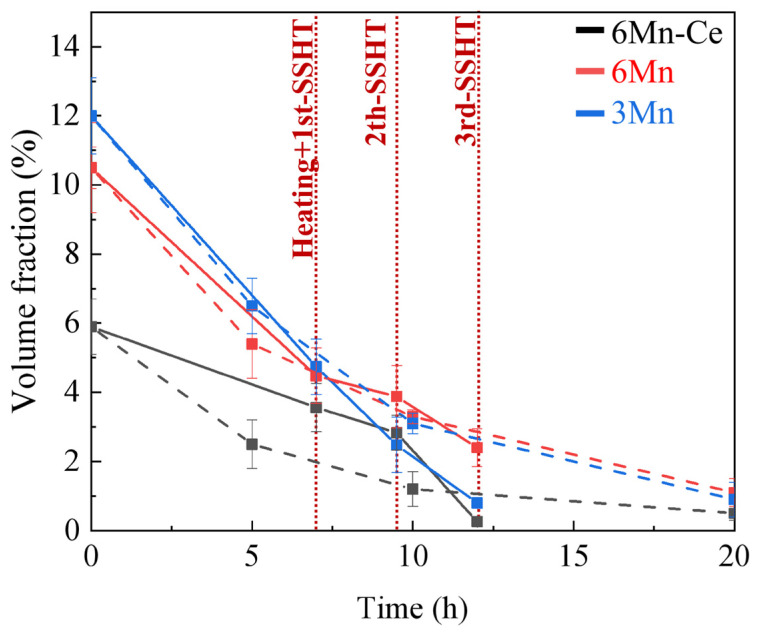
The volume fraction of σ phase under different homogenization processes: the dashed and solid lines are the changes of the volume fraction of σ phase during ISHT and SSHT processes, respectively.

Quantitative statistics were conducted based on the evolution of the σ phase during homogenization, as shown in Figure 9. After 7 h of homogenization (ISHT for 7 h, 1st-SSHT finished), the dissolution rate of the σ phase in 3Mn steels was faster in ISHT, with the residual σ phase about 2.03%, 4.31% and 5.82% in 6Mn-Ce, 6Mn and 3Mn, respectively; meanwhile, the residual σ phase in 6Mn-Ce, 6Mn and 3Mn were 3.56%, 4.48% and 4.74% after SSHT, respectively. After about 9.5 h of homogenization (9.5 h for ISHT and 2nd-SSHT end), 1.16%, 3.01% and 3.88% of the σ phase in 6Mn-Ce, 6Mn and 3Mn remained after ISHT, respectively. While after the 2nd-SSHT, 6Mn-Ce, 6Mn and 3Mn remained the volume fractions of the σ phase of 2.83%, 3.88% and 3.89%, respectively. After 12~13 h of homogenization (ISHT for 12~13 h, end of 3rd-SSHT), the σ phase in 6Mn-Ce, 6Mn and 3Mn was reduced to 0.51%, 2.23% and 2.89% after ISHT, respectively; and the σ phase in 6Mn-Ce, 6Mn and 3Mn was reduced to 0.26%, 2.40% and 0.55% after SSHT, respectively. Therefore, during the ISHT, the dissolution rate of the σ phase first accelerated and then gradually smoothed. While during the SSHT, the dissolution rate was slow at the beginning and then accelerated by higher temperatures. Since the corrosion resistance was almost unaffected when the σ phase was below 0.6% [27], both 6Mn-Ce and 3Mn achieved the homogenization effect under the SSHT.

**Figure 10:**
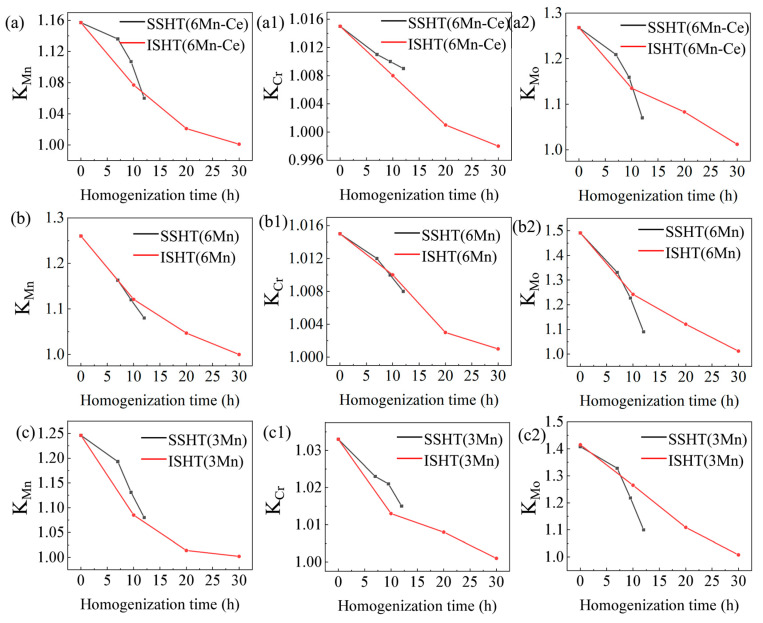
The K value of Mn, Cr and Mo in (**a**–**a2**) 6Mn-Ce, (**b**–**b2**) 6Mn and (**c**–**c2**) 3Mn under ISHT and SSHT.

The elemental distribution during homogenization is illustrated in Figure 10. The red curve represents elemental change during the ISHT, and the black curve is for the SSHT. Figure 10a–c is the 6Mn-Ce, 6Mn and 3Mn steels, respectively. The segregation coefficient is K = K_L_/K_S_, where K_L_ is the alloy contents of inter-dendrites and K_S_ is the element distribution at the dendrites. In general, the further the K value deviates from 1, the greater the segregation. When the 0.95 < K < 1.1, the homogenization is basically completed. 

In the ISHT, the K of each element gradually decreased with the extension of time, and the diffusion rates of Mn, Cr and Mo were faster in the early stage, followed by a smooth trend. Until 30 h, the K value of Cr, Mo and Mn elements reached K ≤ 1.02 which means that the homogenization of the three steels had been completed. For the SSHT, the homogenization rate was relatively slow in the early stage and accelerated when the temperature rises to 1235 °C. After the 3rd-SSHT, the K of all elements of 6Mn-Ce and 3Mn decreased significantly to K < 1.1, reaching the homogenization standard. 

### 3.5. Hardness of the Homogenized Sample

**Figure 11:**
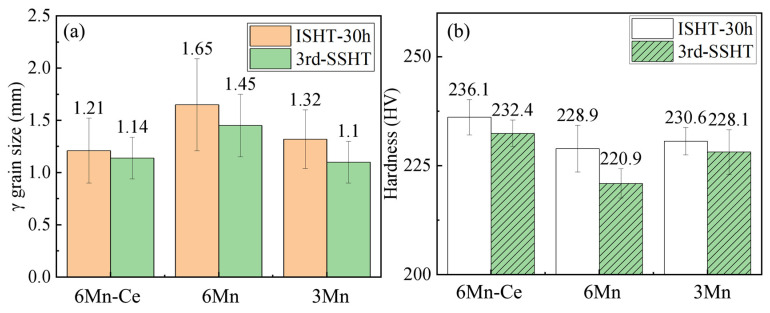
(**a**) the average grain size; and (**b**) the average hardness of 6Mn-Ce,6Mn and 3Mn after ISHT and SSHT.

Figure 11a shows the grain size of 6Mn-Ce, 6Mn and 3Mn after different homogenization processes. After adopting the SSHT, the average austenite grain size of 6Mn-Ce reduced from 1.21 mm to 1.14 mm; the average austenite grain size of 6Mn reduced from 1.65 mm to 1.45 mm; and 3Mn reduced from 1.32 mm to 1.10 mm. This is mainly due to the reduction in holding time at the high temperature. The hardness of the three steels after two homogenization processes was investigated using Vickers hardness, as shown in Figure 11b. It can be seen that using the SSHT can slightly reduce the microstructure hardness, mainly because the “furnace cooling + water quench” reduces the thermal stress rather than the water cooling directly. Due to the amounts of casting defects, such as element segregation and inclusions, furnace cooling was used to reduce the temperature difference, preventing the stress distortion caused by inclusions. On the other hand, the solute elements were fully diffused to avoid internal stress.

The effect of Ce and Mn on the homogenized steels can be noticed according to Figure 11; increasing the Ce content or decreasing the Mn content can reduce the grain size and increase the average hardness. 

## 4. Discussion

By investigating the evolution of the σ phase and element segregation of cast 7Mo SASSs with different Ce and Mn contents during the ISHT and SSHT, the main conclusions are as follows: (1) the SSHT process improves the homogenization efficiency of 6Mn-Ce and 3Mn steels and optimizes the homogenized microstructure; (2) increasing the Ce content or decreasing the Mn content both contribute to a faster homogenization process and improve the properties of the homogenized microstructure. Therefore, the discussion will be further developed based on the above points.

Firstly, the advantages of SSHT are discussed. According to Figure 7d, most of the remelting holes exist at the phase interface. A previous study [29] showed that the dissolved atoms accumulate at the phase interface because of a fast heating rate, which leads to a lower liquidus temperature, triggering overburning. Regarding the remelting region as a semi-solid melting pool, the elements will be segregated at the phase interface as time prolongs [30,31]. At the same time, the liquidus temperature will be lower than 1250 °C, according to Table 3, when the atoms accumulate at phase interfaces. Once the overburning occurs, the σ phase will be formed again from the liquid pool “L + γ + σ” after cooling, making the σ phase hard to be dissolved at the final stage. 

**Figure 12:**
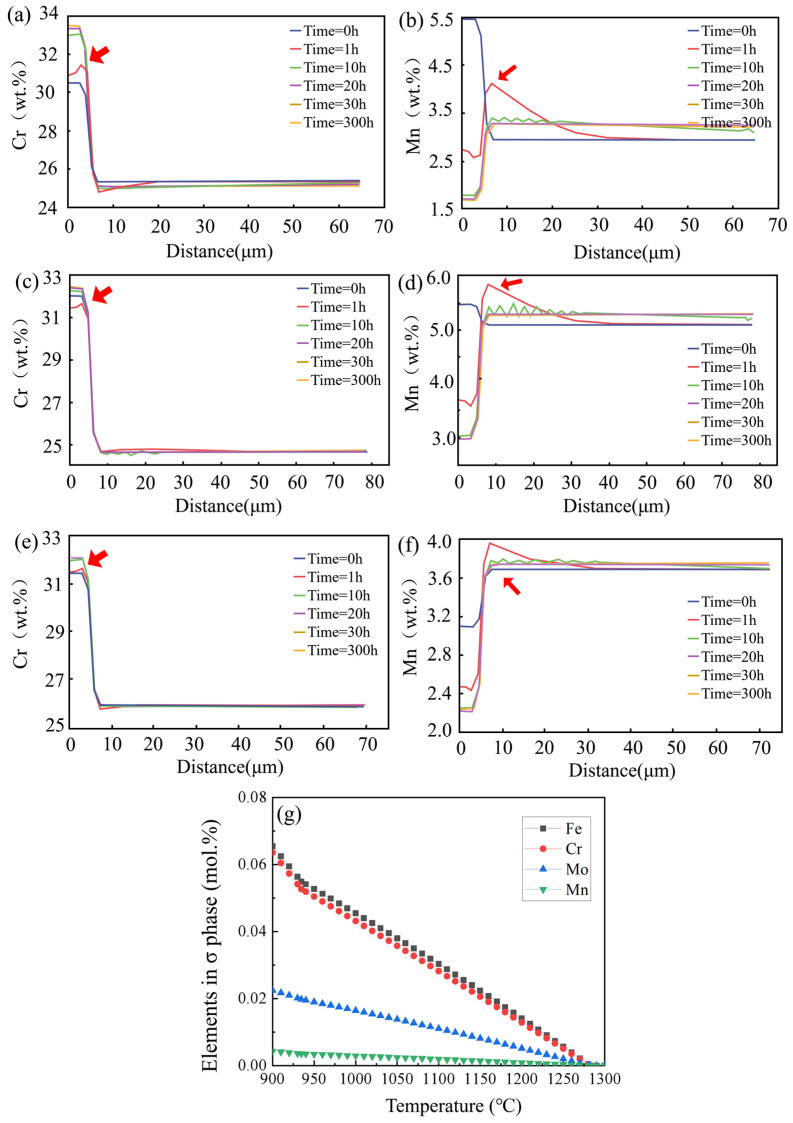
Changes in element distribution at the front of the interface of σ phase during isothermal homogenization: (**a**,**b**) 6Mn-Ce; (**c**,**d**) 6Mn; (**e**,**f**) 3Mn, after 1h ISHT, all elements are enriched at the phase interface, as shown by the arrow; (**g**) variation of σ phase equilibrium composition with temperature in 6Mn-Ce steel.

Moreover, DICTRA was used to calculate the elemental changes at the front of the σ phase interface during the isothermal process, as shown in Figure 12. In the three steels, enrichment of Cr and Mn elements appears at the front of the interface of the σ phase, as shown by the arrow in Figure 12. With the extension of holding time, Mn content in the σ phase decreases, while the Cr element accumulates in the σ phase. For the uphill diffusion within the eutectic phase, it is considered that due to the chemical composition of the eutectic phase deviating from the equilibrium, the elements in the phase will be adjusted spontaneously to lead to the uphill diffusion of alloying elements under long-term isothermal treatment and reduce the homogenization efficiency [32]. The variation of the σ phase in the equilibrium composition of 6Mn-Ce steel with temperature is shown in Figure 12g. In order to ensure the phase stability of the intermetallic compound, the σ phase was adjusted in proportion to the composition element content before reaching the melting point. The SSHT can exactly avoid the long-term isothermal process at a certain temperature and avoid the occurrence of uphill diffusion.

Therefore, for materials with a narrow heat treatment temperature range, the SSHT is more suitable. The slow heating rate can effectively reduce the temperature fluctuation of the furnace and increase the diffusion coefficient of the atoms, which accelerates the diffusion of elements that are produced by the dissolved σ phase [19]. The application of graded insulation is intended to increase the liquidus temperature step by step. This method is also verified in CMSX-10 [33], where the graded insulation helps to increase the liquidus temperature by 25 °C. 

The effects of Ce and Mn elements on the homogenization process can be obtained from Figure 7 and Figure 8. According to the slope of the dissolution curve of the σ phase in 6Mn-Ce, 6Mn and 3Mn, the dissolution rate of the σ phase obeys the relationship of 6Mn-Ce > 3Mn > 6Mn, which shows the dissolution of the σ phase is accelerated by increasing the Ce or Mn content. A study denoted that decreasing dendrite spacing or reducing the initial concentration can both accelerate the diffusion process [34]. The addition of Ce promotes the formation of rare-earth inclusions in SASSs [3,8]. These inclusions can refine the dendrites to reduce the second phase, which also can play a role in pinning grain boundaries at high temperatures to increase the average hardness. However, excess Mn contents will participate in the formation of Ce-O-Mn inclusions with little effect on heterogeneous nucleation, resulting in coarse dendrites [7], leading to a lower dissolution rate. Meanwhile, according to Figure 8, the dissolution rate of Cr and Mo elements slows down at the initial stage of homogenization when the Mn content is increased from 3 wt.% to 5 wt.%. According to the previous study, the Mn element has the effect of promoting the dendritic segregation of Cr and Mo elements, which is the main reason for the lower dissolution rate.

## 5. Conclusions

In this study, the microstructural evolution of 7Mo super austenitic stainless steels with different Ce and Mn contents during homogenization were investigated, and the effect of alloy elements and homogenization parameters on the dissolution process were explored by studying dissolution kinetics and hardness, with the following results:

(1) Increasing the Ce element can refine the cast microstructure and reduce the σ phase from 10.5% to 5.9%, which speeds up the homogenization process and increases the average hardness of the homogenized microstructure.
(2) Raising the Mn element promotes severe element segregation and coarse dendrites in the cast microstructures, which slows down the dissolution rate of atoms. Homogenized grains are also enlarged, with smaller hardness after the Mn contents increase by 2 wt.%.
(3) The stepped solution heat treatment can make the cast 7Mo SASS meet the homogenization standard efficiently, shorten the homogenization time from 30 h to 12~13 h, as well as avoid the incipient remelting.

## Figures and Tables

**Table 1:** Chemical composition of 7Mo SASSs (wt.%).

	Cr	Mo	Ni	Mn	N	Cu	Si	Ce	Fe
6Mn-Ce	25.012	6.771	18.891	5.733	0.459	0.405	0.091	0.024	bal.
6Mn	25.182	6.892	18.819	6.028	0.418	0.412	0.108	0.001	bal.
3Mn	25.321	7.088	19.052	3.124	0.438	0.443	0.113	0.001	bal.

**Table 2:** Temperatures of completely dissolved σ phase (T_σ_); temperatures at which the δ phase (T_δ_) and the liquid (T_L_) formed.

	T_σ_ (°C)	T_δ_ (°C)	T_L_ (°C)
6Mn-Ce	1267.48	1268.00	1326.21
6Mn	1267.82	1268.30	1326.18
3Mn	1279.10	1285.84	1338.00

**Table 3:** Calculation of the liquidus temperature (T_LL_).

wt.%	Cr	Mo	Mn	Ni	Fe	Ce	T_LL_ (°C)
6Mn-Ce	25.66	6.62	6.42	16.16	42.00	0.02	1250.74
6Mn	25.05	8.55	6.43	17.01	39.69	0.001	1248.30
3Mn	25.94	7.51	3.68	17.84	41.68	0.001	1264.38

## Data Availability

All data are reported in the article.
